# State Abortion Policy and Moral Distress Among Clinicians Providing Abortion After the *Dobbs* Decision

**DOI:** 10.1001/jamanetworkopen.2024.26248

**Published:** 2024-08-01

**Authors:** Katherine Rivlin, Marta Bornstein, Jocelyn Wascher, Abigail Norris Turner, Alison H. Norris, Dana Howard

**Affiliations:** 1 Department of Obstetrics and Gynecology, University of Chicago Medicine, Chicago, Illinois; 2 Department of Health Promotion Education and Behavior, Arnold School of Public Health, University of South Carolina, Columbia; 3 Department of Obstetrics and Gynecology, University of Chicago Medicine, Chicago, Illinois; 4 College of Public Health, The Ohio State University, Columbus; 5 Division of Epidemiology, Colleges of Public Health and Medicine, The Ohio State University, Columbus; 6 Division of Bioethics, Department of Biomedical Education and Anatomy, The Ohio State University College of Medicine, Columbus

## Abstract

**Importance:**

Moral distress occurs when individuals feel powerless to do what they think is right, including when clinicians are prevented from providing health care they deem necessary. The loss of federal protections for abortion following the *Dobbs v Jackson Women’s Health Organization* Supreme Court decision may place clinicians providing abortion at risk of experiencing moral distress, as many could face new legal and civil penalties for providing care in line with professional standards and that they perceive as necessary.

**Objective:**

To assess self-reported moral distress scores among abortion-providing clinicians following the *Dobbs* decision overall and by state-level abortion policy.

**Design, Setting, and Participants:**

This survey study, conducted from May to December 2023, included US abortion-providing clinicians (physicians, advanced practice clinicians, and nurses). A purposive electronic survey was disseminated nationally through professional listservs and snowball sampling.

**Exposure:**

Abortion policy in each respondent’s state of practice (restrictive vs protective using classifications from the Guttmacher Institute).

**Main Outcomes and Measures:**

Using descriptive statistics and unadjusted and adjusted negative binomial regression models, the association between self-reported moral distress on the Moral Distress Thermometer (MDT), a validated psychometric tool that scores moral distress from 0 (none) to 10 (worst possible), and state abortion policy was examined.

**Results:**

Overall, 310 clinicians (271 [87.7%] women; mean [SD] age, 41.4 [9.7] years) completed 352 MDTs, with 206 responses (58.5%) from protective states and 146 (41.5%) from restrictive states. Reported moral distress scores ranged from 0 to 10 (median, 5) and were more than double for clinicians in restrictive compared with protective states (median, 8 [IQR, 6-9] vs 3 [IQR, 1-6]; *P* < .001). Respondents with higher moral distress scores included physicians compared with advanced practice clinicians (median, 6 [IQR, 3-8] vs 4 [IQR, 2-7]; *P* = .005), those practicing in free-standing abortion clinics compared with those practicing in hospitals (median, 6 [IQR, 3-8] vs 4 [IQR, 2-7]; *P* < .001), those no longer providing abortion care compared with those still providing abortion care (median, 8 [IQR, 4-9] vs 5 [IQR, 2-8]; *P* = .004), those practicing in loss states (states with the greatest decline in abortion volume since the *Dobbs* decision) compared with those in stable states (unadjusted incidence rate [IRR], 1.72 [95% CI, 1.55-1.92]; *P* < .001; adjusted IRR, 1.59 [95% CI, 1.40-1.79]; *P* < .001), and those practicing in surge states (states with the greatest increase in abortion volume since the *Dobbs* decision) compared with those in stable states (unadjusted IRR, 1.27 [95% CI, 1.11-1.46]; *P* < .001; adjusted IRR, 1.24 [95% CI, 1.09-1.41]; *P* = .001).

**Conclusions and Relevance:**

In this purposive national survey study of clinicians providing abortion, moral distress was elevated among all clinicians and more than twice as high among those practicing in states that restrict abortion compared with those in states that protect abortion. The findings suggest that structural changes addressing bans on necessary health care, such as federal protections for abortion, are needed at institutional, state, and federal policy levels to combat widespread moral distress.

## Key Points

**Question:**

Do clinicians providing abortion practicing in states that restrict abortion experience more moral distress than those practicing in states that protect abortion?

**Findings:**

In this national, purposive survey study of 310 clinicians providing abortion, moral distress was elevated among all clinicians, with those practicing in restrictive states reporting higher levels of moral distress compared with those practicing in protective states.

**Meaning:**

The findings suggest that structural changes addressing bans on necessary health care, such as federal protection for abortion, are needed at institutional, state, and federal policy levels to address clinician moral distress.

## Teaser

This survey study assesses moral distress scores among abortion-providing clinicians practicing in states with restrictive vs protective abortion policies following the *Dobbs v Jackson Women’s Health Organization* Supreme Court decision.

## Introduction

The US Supreme Court’s decision on *Dobbs vs Jackson Women’s Health Organization* eliminated federal protections for abortion, leaving states with independent regulatory authority. Since the decision, abortion is almost entirely banned in 14 US states. In at least 11 additional states, abortion is severely restricted.^1^ Clinicians could face new legal and civil penalties for providing abortion, including felony charges and loss of medical license.^2^ In many states with abortion bans, the list of exceptions is narrow and confusing, with few or no exceptions for maternal health or life endangerment.^3,4^ In this context, clinicians may increasingly find themselves facing a difficult dilemma: either fail to provide appropriate, conscience-driven medical care or put themselves in legal and professional jeopardy. This conflicting experience may give rise to moral distress, or the emotional harm that occurs when a clinician’s conscience guides them to provide specific care that is in line with professional standards, but external constraints, such as state or institutional policies, block them from being able to do so.^5,6,7,8^

The concept of moral distress in health care originates from discussions with nurses over care that they are expected to provide but they ethically oppose.^9,10^ While early definitions of moral distress focused on negative claims of conscience (or when individuals object to care required of them, such as overtreatment during end of life), the definition has since broadened to include positive claims of conscience, or the inability to provide the care that one feels morally compelled to provide. In this context, moral distress has direct applications to abortion-providing clinicians facing abortion bans.^11,12^ Prolonged exposure to moral distress without resolution can lead to moral injury. Both moral distress and moral injury have been associated with clinician burnout, psychological distress, and low self-reported well-being.^13,14,15,16^

Since the *Dobbs* decision, commentaries and qualitative studies have raised concerns about increasing moral distress among clinicians.^6,7,17^ We aimed to quantify moral distress among clinicians providing abortion following the *Dobbs* decision and to assess differences by state-level abortion policy. We hypothesized that clinicians in states that restrict abortion would report higher moral distress scores compared with clinicians in states that protect abortion. We also aimed to identify additional factors that are positively or negatively associated with moral distress.

## Methods

### Study Setting and Participant Recruitment

This survey study followed the American Association for Public Opinion Research (AAPOR) reporting guideline.^18^ The institutional review board at The Ohio State University approved this study. Electronic informed consent was obtained before completion of the anonymous, online survey. Participants who completed the survey were eligible to receive a $10 gift card.

From May to December 2023, we recruited clinicians (physicians, advanced practice clinicians, and nurses) whose practice included abortion care to complete a 30-item online survey querying personal demographics, practice characteristics including state of practice, and the experience of moral distress related to their abortion care practice (eAppendix in Supplement 1). We included clinicians providing abortion care at the time of the survey and those not currently providing but who had provided abortion care between May 2021 and June 2022 (the year prior to the *Dobbs* decision). We excluded nonclinicians and clinicians who reported providing abortion care neither at the time of the survey nor in the year prior to the *Dobbs* decision.

We recruited participants through email using professional listservs tailored toward clinicians who provide abortion. We also used snowball sampling by encouraging respondents to forward recruitment materials to their colleagues who could meet inclusion criteria. Prospective participants completed an electronic prescreening eligibility questionnaire.

Moral distress could be associated with race and ethnicity^6^; therefore, we asked participants to self-report these variables. Race categories included African American or Black, Asian, White, and multiple races selected. Ethnicity categories were Hispanic or Latinx and not Hispanic or Latinx.

### Sample Size

The number of individual clinicians providing abortion in the US is challenging to measure. In their 2020 Abortion Provider Census, the Guttmacher Institute (a research and policy organization that uses evidence to advance sexual and reproductive health) identified 1603 health care facilities nationally that provided abortion but did not assess the number of individual clinicians.^19,20^ Given these limitations on defining a sampling frame, our recruitment goal was more general: to collect a purposive sample of abortion-providing clinicians, including respondents from states with both low and high numbers of abortion clinics. Using the 2020 Abortion Provider Census, we divided states into low clinic number (<15 clinics statewide) and high clinic number (≥15 clinics statewide). Around 80% of abortion clinics were in states with high clinic numbers. To capture a wider range of experiences, we aimed to oversample clinicians from states with a low clinic number, with a plan to continuously assess and target recruitment such that the sample composition remained at or above 20% of respondents from states with a low clinic number.

### Dependent Variables

#### Moral Distress Thermometer

**Figure 1.**
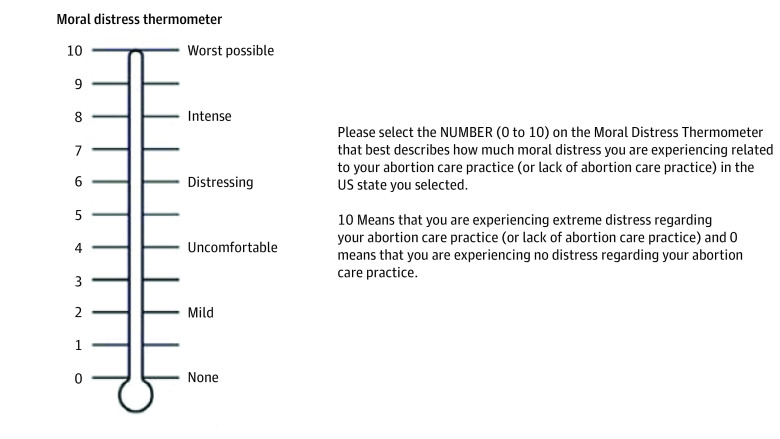
Moral Distress Thermometer and Prompt Derived from Wocial and Weaver.^21^

We examined moral distress as our dependent variable. All participants completed a modified version of the Moral Distress Thermometer (MDT), a single-item scale to measure moral distress. Scores range from 0 (none) to 10 (worst possible), with written descriptors to anchor degree of distress (Figure 1). The MDT is a validated psychometric screening tool originally used to measure moral distress among nurses in hospital settings and since used in multiple health care contexts.^21,22,23^ Our modification to the instrument is consistent with instrument construction: it includes the original definition and rating scale and asks respondents to reflect on their clinical practice in a specific context.^21^

#### Self-Reported Changes in Moral Distress After Compared With Before the *Dobbs* Decision

In addition to the MDT, the survey asked clinicians to report whether they had experienced more, less, or the same levels of moral distress after compared with before the *Dobbs* decision. We analyzed these responses to understand self-reported changes in moral distress among all respondents.

### Primary Independent Variables

The first independent variable that we examined was whether a respondent’s reported US state of abortion care practice was restrictive or protective according to the Guttmacher Institute’s classification (state policy category).^1^ To assess state of abortion practice, we asked clinicians to select 1 US state of practice to keep in mind when answering questions related to moral distress. Because abortion-providing clinicians can practice in more than 1 state, we allowed participants to provide moral distress responses for up to 3 US states. We used the Guttmacher Institute’s abortion policy categories as of December 2023 (when the survey closed) to characterize each state. The Guttmacher classification groups US states into 1 of 7 categories, from “most restrictive” to “most protective,” based on abortion policies currently in effect.^1^ We collapsed the 7 categories into 2: restrictive (includes “most restrictive,” “very restrictive,” and “restrictive”) and protective (includes “most protective,” “very protective,” and “protective”). We included the middle category “some restrictions/protections” in the protective category.

The second independent variable that we examined was whether a respondent practiced in a state with a large change in abortion volume after the *Dobbs* decision, classified as a surge, loss, or stable (neither surge nor loss) state. We constructed these categories based on WeCount, a national abortion reporting effort.^24^ WeCount defines surge states as those with the largest cumulative increase in total number of abortions provided in the 12-month period following the *Dobbs* decision (Illinois, Florida, North Carolina, California, and New Mexico) and defines loss states as those with the largest decline in total number of abortions during the 12-month period following the *Dobbs* decision (Texas, Georgia, Louisiana, Wisconsin, and Alabama).^24^ We examined associations of surge, loss, and stable state classification both with and without California given that California is a surge state but experienced only a 4% increase in overall abortion volume while other surge states experienced an 18%-32% increase in abortion volume following the *Dobbs*, as changes in relative volume may drive clinician experiences of moral distress.

### Statistical Analysis

We first assessed respondent moral distress scores descriptively overall and by our main independent variables, demographic characteristics, and abortion practice characteristics. We assessed differences in median moral distress using Kruskal-Wallis tests (Table 1).

**Table 1.** Median MDT Scores by Participant Characteristics

Variable	MDTs, No. (%)	MDT score, median (IQR)	*P* value^a^
Total	352 (100)	5 (2-8)	NA
**Primary independent variables**			
Policy context			
Protective or both restrictions/protections	206 (58.5)	3 (1-6)	<.001
Most restrictive, very restrictive, restrictive	146 (41.5)	8 (6-9)	
Surge or loss state			
Neither	219 (62.2)	5 (2-7)	<.001
Surge	101 (28.7)	5 (2-8)	
Loss	32 (9.1)	8 (8-10)	
Surge or loss state excluding CA (n = 318)			
Neither	219 (68.9)	5 (2-7)	<.001
Surge	67 (21.1)	7 (5-8)	
Loss	32 (10.1)	8 (8-10)	
**Demographic characteristics**			
Age, y (n = 344)			
27-35	106 (30.8)	5 (2-8)	.86
36-45	146 (42.4)	6 (3-8)	
46-55	57 (16.6)	5 (3-8)	
56-78	35 (10.2)	7 (1-8)	
Missing	8		
Race (n = 343)			
African American or Black	7 (2.0)	7 (5-10)	.38
Asian	22 (6.4)	6 (3-8)	
Multiple races selected	17 (5.0)	5 (2-8)	
White	297 (86.6)	5 (2-8)	
Missing	9	NA	
Ethnicity (n = 349)			
Hispanic or Latinx	31 (8.9)	4 (2-8)	.66
Non-Hispanic or Latinx	318 (91.1)	6 (2-8)	
Missing	3		
Gender identity (n = 351)			
Man	28 (8.0)	7 (4-8)	.45
Nonbinary, gender fluid, or agender	15 (4.3)	5 (2-8)	
Woman	308 (87.8)	5 (2-8)	
Missing	1	NA	
**Abortion practice characteristics**			
Health care role			
Physician	254 (72.2)	6 (3-8)	.005
Nurse	25 (7.1)	5 (1-8)	
Advanced practice clinician	73 (20.7)	4 (2-7)	
Physician specialty (physician only; n = 250)			
OBGYN	180 (72.0)	6 (3-8)	.62
Family medicine	70 (28.0)	6 (3-8)	
Missing	4	NA	
Complex family planning fellowship (physician only; n = 254)			
Completed or currently completing	126 (49.6)	6 (3-8)	.19
No	128 (50.4)	6 (4-8)	
Currently in training			
Yes	28 (8.0)	6 (2-8)	.77
No	324 (92.0)	5 (2-8)	
Time providing abortion care, y			
0.5-5	112 (31.8)	5 (2-8)	.31
6-10	108 (30.7)	6 (3-8)	
11-20	90 (25.6)	5 (3-7)	
>20	42 (11.9)	7 (3-8)	
Currently providing abortion			
Yes	330 (93.8)	5 (2-8)	.004
No	22 (6.3)	8 (4-9)	
Proportion of practice focused on abortion, % (n = 351)			
0	22 (6.3)	8 (4-9)	.01
1-50	208 (59.3)	5 (2-8)	
51-100	121 (34.5)	6 (2-8)	
Missing	1	NA	
Current practice or pre*-Dobbs* setting (n = 351)			
Hospital or non–free-standing abortion clinic	131 (37.3)	4 (2-7)	<.001
Free standing abortion clinic, including Planned Parenthood	220 (62.7)	6 (3-8)	
Missing	1	NA	
Region of practice			
West	69 (19.6)	3 (1-5)	<.001
Southwest	34 (9.7)	8 (6-10)	
Midwest	77 (21.9)	6 (4-8)	
Southeast	89 (25.3)	7 (5-8)	
Northeast	83 (23.6)	3 (1-5)	

Abbreviations: MDT, Moral Distress Thermometer; NA, not applicable; OBGYN, obstetrics and gynecology.

^a^ *P* values are for Kruskal-Wallis tests comparing differences in medians.

We then constructed unadjusted and adjusted negative binomial regression models to estimate associations between (1) state policy category (restrictive or protective) and MDT scores and (2) post-*Dobbs* state change in abortion volume category (surge, loss, or stable) and MDT scores. We accounted for clustering by respondent, as participants could complete MDTs for multiple states of practice. We selected negative binomial regression models to address observed overdispersion in the outcome, a count variable (MDT score).

In adjusted models, we included covariates that may be associated with either state policy category or state change in abortion volume category and with MDT score. These included (1) role in health care (physician, nurse, or advanced practitioner), as state laws can prohibit advanced practice clinicians from providing abortion care; (2) health care setting (free-standing abortion clinic vs other), as state laws can restrict hospital-based abortion care, leaving the majority of abortion care to occur in free-standing clinics; and (3) whether respondents were providing abortion following the *Dobbs* decision (yes, no), given that some states have banned abortion.

We conducted 2 sensitivity analyses altering how we coded state policy category to assess the robustness of our findings. First, during the time of the survey, the Guttmacher Institute’s categorization of some state abortion policy changed. Kansas moved from restrictive to protective. We reanalyzed the data with Kansas as protective. Second, we moved the Guttmacher category “some restrictions/protections” into the restrictive category.

We conducted all analyses using Stata, version 18 (StataCorp LLC). A priori, we set the type 1 error rate (α) to 0.05, and declared *P* < .05 to be statistically significant.

## Results

### Sample Characteristics

We do not know how many individuals received the survey, as we recruited through listservs that protect their members’ identities and through snowball sampling. Of 388 respondents who began the survey, 346 met eligibility criteria and provided consent (89.2%). For this analysis, we included only eligible and consenting respondents who provided complete MDT responses (310 [89.6%]). Of these 310 respondents, 25 (8.1%) were men, 271 (87.7%) were women, and 13 (4.2%) were nonbinary, gender fluid, or agender (1 respondent was missing gender data). Mean (SD) age was 41.4 (9.7) years. A total of 6 (2.0%) were African American or Black; 21 (6.8%), Asian; 262 (84.5%), White; and 15 (4.8%) selected multiple races (6 people selected other, don’t know, or prefer not to answer). A total of 25 (8.1%) were Hispanic or Latinx, and 282 (91.9%) were not Hispanic or Latinx (3 respondents were missing ethnicity data). These respondents completed 352 MDTs for states of practice, with 37 respondents completing MDTs for 2 states and 5 respondents completing MDTs for 3 states. Of those who consented but did not provide MDTs (36 total), 18 did not provide state of practice. Of the 18 respondents who provided state of practice but no MDT, 5 (27.8%) practiced in at least 1 restrictive state. We have no demographic information on respondents not meeting eligibility or providing consent.

The sample included respondents providing MDT responses for abortion practices in 43 US states and Washington, DC, with 116 MDTs (33.0%) coming from states with a low abortion clinic number. We did not perform additional recruitment in these states as our sample always remained greater than 20% from these states. Over half of MDT responses (206 [58.5%]) were for states protecting abortion, and 146 (41.5%) were for states restricting abortion. Most respondents were physicians (222 [71.6%]). Of these physicians, 159 (71.6%) were in obstetrics and gynecology; 59 (26.6%), family medicine; 114 (51.4%), complex family planning fellowship trained; and 4 (1.8%), other type of physician. The remaining respondents included 66 advanced practice clinicians (21.3%) and 22 nurses (7.1%). Only 21 respondents (6.8%) had practices composed of only abortion care, with the remainder (288 [92.9%]) also providing nonabortion health care. Trainees represented 8.4% of the sample (n = 26).

### Moral Distress Thermometer

**Figure 2.**
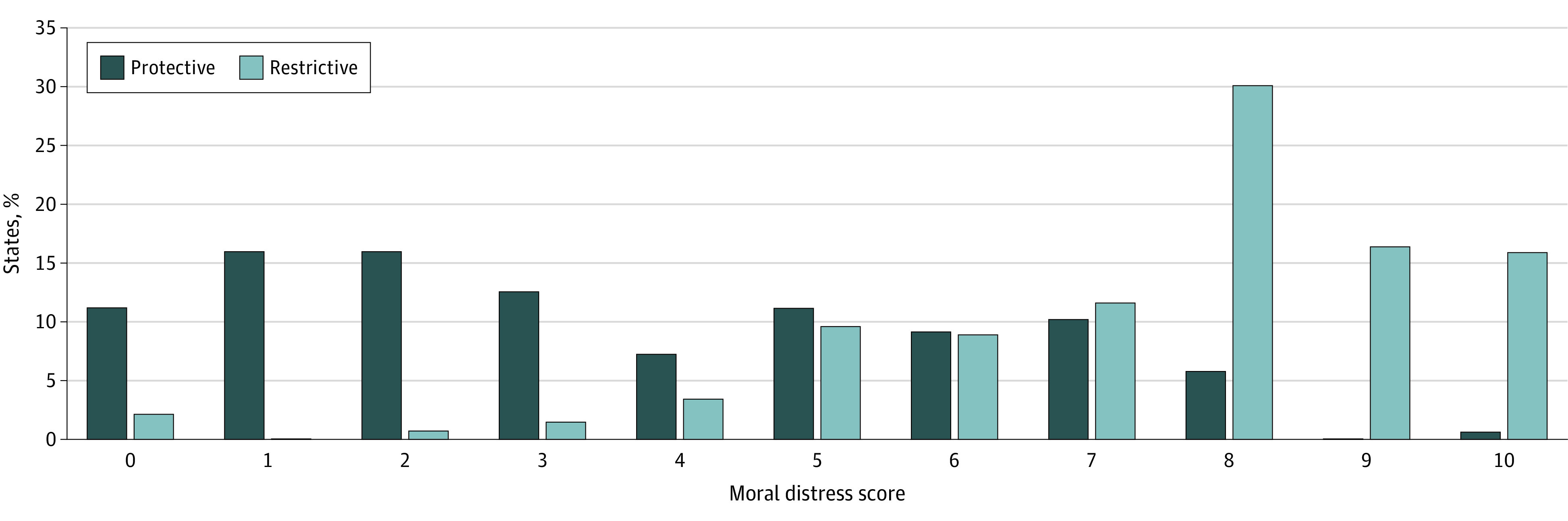
Distribution of Median Moral Distress Thermometer Scores by State Abortion Policy A total of 352 Moral Distress Thermometers were completed by 310 clinicians.

Moral Distress Thermometer scores ranged from 0 to 10 (median, 5, [IQR, 2-8], or between “uncomfortable” and “distressing”) and were more than twice as high for clinicians practicing in restrictive states compared with those practicing in protective states (median, 8 [IQR, 6-9], or “intense” vs 3 [IQR, 1-6], or between “mild” and “uncomfortable”; *P* < .001) (Table 1 and Figure 2). Moral Distress Thermometer scores were highest in loss states compared with surge and stable states (8 [IQR, 8-10] vs 5 [IQR, 2-8] vs 5 [IQR, 2-7]; *P* < .001). Clinicians in surge states had higher MDT scores compared with clinicians in stable states (median, 7 [IQR, 5-8] vs 5 [IQR, 2-7]; *P* < .001) (excluding California) (Table 1).

**Figure 3.**
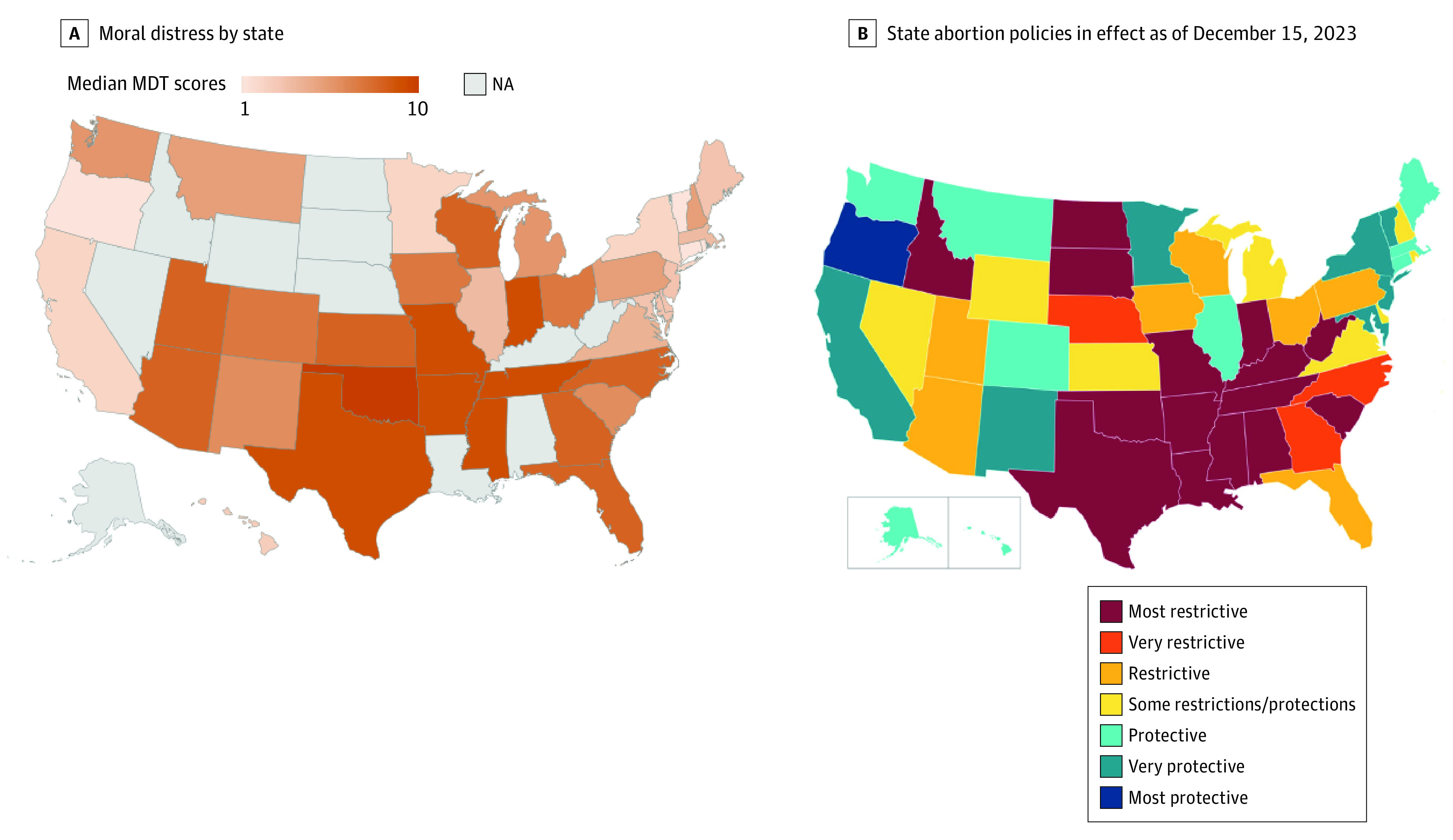
Median Moral Distress Thermometer (MDT) Score by State With Guttmacher Institute Abortion Policy Map B, Based on “Interactive Map: US Abortion Policies and Access After Roe,” Guttmacher Institute, guttmacher.org.

Moral Distress Thermometer scores were higher for physicians compared with advanced practice clinicians (median, 6 [IQR, 3-8] vs 4 [IQR, 2-7]; *P* = .005) and for those practicing in free-standing clinics (eg, Planned Parenthood or independent abortion clinics) compared with those in other practice settings such as hospitals (median, 6 [IQR, 3-8] vs 4 [IQR, 2-7]; *P* < .001). Moral Distress Thermometer scores for respondents no longer providing abortion care were higher compared with MDT scores for those still providing care (8 [IQR, 4-9] vs 5 [IQR, 2-8]; *P* = .004) (Table 1). Moral Distress Thermometer scores were highest in the Southwest (median, 8 [IQR, 6-10]) and lowest in the West (median, 3 [IQR, 1-5]) and Northeast (median, 3 [IQR, 1-5]) (*P* < .001) (Table 1 and Figure 3).

### Self-Reported Changes in Moral Distress After Compared With Before the *Dobbs* Decision

For each MDT, the majority of respondents (275 [78.1%]) reported experiencing more moral distress since the *Dobbs* decision. For only 15 MDTs (4.3%), respondents reported experiencing less moral distress.

### Association Between State-Level Policy and Moral Distress

In unadjusted regression analyses, moral distress was higher among clinicians in restrictive states compared with those in protective states (unadjusted incidence rate ratio [IRR], 2.14; 95% CI, 1.92-2.40; *P* < .001). After adjusting for health care role, whether currently providing abortion, and practice setting, the results did not change meaningfully (adjusted IRR, 2.03; 95% CI, 1.81-2.83; *P* < .001) (Table 2). In sensitivity analyses, the results did not change meaningfully from the primary analyses when recategorizing Kansas from restrictive to protective (adjusted IRR, 1.91; 95% CI, 1.71-2.15; *P* < .001) and moving “some restrictions/protections” from the protective to the restrictive category (adjusted IRR, 2.01; 95% CI, 1.78-2.28; *P* < .001).

**Table 2.** Unadjusted and Adjusted Negative Binomial Models Examining the Association of Abortion Policy Context and Change in Abortion Volume Since the *Dobbs* Decision With Levels of Moral Distress

Variable	Unadjusted		Adjusted	
	IRR (SE) [95% CI]	*P* value	IRR (SE) [95% CI]	*P* value
**Model A (n = 351)** ^a^				
Policy context				
Protective or both restrictions/protections	1 [Reference]	NA	1 [Reference]	NA
Most restrictive, very restrictive, or restrictive	2.14 (0.12) [1.92-2.40]	<.001	2.03 (0.12) [1.81-2.83]	<.001
Health care role				
Physician	1 [Reference]	NA	1 [Reference]	NA
Nurse	0.85 (0.15) [0.59-1.21]	.36	0.80 (0.11) [0.61-1.06]	.12
Advanced practice clinician	0.76 (0.07) [0.63-0.92]	.005	0.88 (0.07) [0.76-1.03]	.12
Currently providing abortion				
Yes	1 [Reference]	NA	1 [Reference]	NA
No	1.37 (0.14) [1.13-1.67]	.001	1.23 (0.08) [1.07-1.40]	.002
Current practice or pre-*Dobbs* setting				
Hospital or non–free-standing abortion clinic	1 [Reference]	NA	1 [Reference]	NA
Free-standing abortion clinic	1.38 (0.10) [1.19-1.59]	<.001	1.14 (0.07) [1.01-1.29]	.04
**Model B (n = 317)** ^b^				
Surge or loss state				
Neither	1 [Reference]	NA	1 [Reference]	NA
Surge	1.27 (0.09) [1.11-1.46]	<.001	1.24 (0.08) [1.09-1.41]	.001
Loss	1.72 (0.09) [1.55-1.92]	<.001	1.59 (0.10) [1.40-1.79]	<.001
Health care role				
Physician	1 [Reference]	NA	1 [Reference]	NA
Nurse	0.82 (0.15) [0.57-1.16]	.26	0.81 (0.13) [0.59-1.11]	.20
Advanced practice clinician	0.83 (0.08) [0.68-1.00]	.053	0.81 (0.08) [0.68-0.98]	.027
Currently providing abortion				
Yes	1 [Reference]	NA	1 [Reference]	NA
No	1.31 (0.13) [1.08-1.60]	.007	1.14 (1.12) [0.93-1.40]	.21
Current practice or pre-*Dobbs* setting				
Hospital or non–free-standing abortion clinic	1 [Reference]	NA	1 [Reference]	NA
Free-standing abortion clinic	1.36 (0.10) [1.18-1.58]	<.001	1.33 (0.10) [1.15-1.53]	<.001

Abbreviations: IRR, incidence rate ratio; NA, not applicable.

^a^ Association between abortion policy context and level of moral distress; clustered at the individual level.

^b^ Association of change in abortion volume since the *Dobbs* decision with level of moral distress; clustered at the individual level. Excludes surveys from California.

### Association Between Post-*Dobbs* Change in Abortion Volume and Moral Distress

Moral distress among clinicians in loss states was higher than moral distress among clinicians in stable states in both unadjusted and adjusted models (unadjusted IRR, 1.72 [95% CI, 1.55-1.92]; *P* < .001; adjusted IRR, 1.59 [95% CI, 1.40-1.79]; *P* < .001). Moral Distress Thermometer scores in surge states were also higher than in stable states (unadjusted IRR, 1.27 [95% CI, 1.11-1.46]; *P* < .001; adjusted IRR, 1.24 [95% CI, 1.09-1.41]; *P* = .001) (Table 2).

## Discussion

This study found that since the *Dobbs* decision, a sample of clinicians providing abortion nationally have experienced median moral distress scores between “uncomfortable” and “distressing” on the MDT. Clinicians in states with a restrictive abortion policy reported MDT scores more than twice as high as clinicians in states with a protective policy (“intense” vs between “mild” and “uncomfortable”). Most MDTs (78.1%) indicated that abortion-providing clinicians experienced more moral distress after compared with before the *Dobbs* decision. Factors positively associated with moral distress included being a physician, practicing in free-standing abortion clinics, no longer providing abortion care since the *Dobbs* decision, practicing in loss or surge states, and practicing in the Southwest, Southeast, or Midwest.

To our knowledge, this study is the first to quantitatively measure moral distress among clinicians providing abortion in any context, including since the *Dobbs* decision; to compare moral distress across state policy environments; and to include midlevel clinicians and nurses. Our study’s quantitative outcomes compliment the results of the qualitative study by Sabbath et al^17^ that described the experience of 54 obstetrician-gynecologists practicing under abortion bans. In that study, among interviewees, 93% reported moral distress, or the experience of “not follow[ing] clinical standards due to legal constraints.”

In our study, individuals no longer providing abortion care and individuals practicing in loss states had higher moral distress scores compared with those still providing abortion care and those practicing in surge or stable states, respectively. Losing one’s professional identity has been similarly associated with moral distress in other health care contexts.^25,26,27^ Individuals who previously provided abortion and who may continue to interact with patients seeking abortion care that they can no longer provide may uniquely experience moral distress or moral injury.

Our findings indicated elevated moral distress among some clinicians in protective states. Such clinicians may experience uncertainty regarding the national abortion legal climate, fears for patients and colleagues in restrictive states, and institutional rather than state restrictions. We also found high moral distress among clinicians in surge states (states often categorized as protective), which may reflect increasing patient volumes, resource scarcity, and witnessing pregnant patients traveling from out of state after being prevented from receiving necessary medical care. Moral distress has been identified in similar health care contexts, including during the COVID-19 pandemic when health care workers faced worsening outcomes, resource shortages, and lack of supportive policies.^11,28^

Some may argue that moral distress could be elevated in abortion-providing clinicians at baseline. However, in a previous survey, abortion-providing clinicians reported higher compassion satisfaction, or pleasure from doing their job well, compared with other health care clinicians.^29^ High compassion satisfaction indicates high levels of pride in the work that one provides. Moreover, regardless of baseline, we found that most clinicians in this survey study reported feeling more moral distress after compared with before the *Dobbs* decision.

Clinicians who are members of racially and ethnically marginalized communities may be at particular risk of moral distress.^6^ While we found no association between race or ethnicity and MDT scores, the sample included few Black and Hispanic or Latinx respondents. Similarly, previous commentaries have focused on moral distress among trainees.^5,6^ We found no such association, although the sample included few trainees.

### Implications

Given its association with burnout and job attrition, prolonged moral distress could drive abortion clinicians to leave the workforce.^14,15,16^ With most of the sample (93.2%) providing some nonabortion-related health care, clinician attrition has implications for the broader maternal health workforce. Before the *Dobbs* decision, states that restricted abortion had higher rates of maternal mortality compared with those that did not.^30^ If moral distress contributes to reproductive health care clinician attrition in abortion-restrictive states, clinician shortages in these states could grow, widening state-level disparities in pregnancy-related mortality.

Recent commentaries have proposed encouraging compassion to mitigate the effects of moral distress among clinicians since the *Dobbs* decision.^6^ Compassion and resilience training have proven to be successful interventions to reduce moral distress in other health care contexts.^31^ Before the *Dobbs* decision, abortion-providing clinicians engaged in such practices, including by distancing themselves from state-mandated language to create emotional alignment with patients and by providing conscientious care in the presence of barriers.^32,33,34^ However, our findings indicate the need to look beyond expecting individuals to build compassion and resilience given the widespread nature of moral distress in the abortion-providing workforce.

### Limitations

This study has limitations. Our survey carried the risk of selection bias, as clinicians responding to our survey may have been more likely to have experienced moral distress compared with those not participating. To our knowledge, no pre-*Dobbs* assessment of moral distress among abortion-providing clinicians exists for comparison. We could not calculate our response rate given the challenges in defining the sample population, the use of recruitment through listservs that protect members’ identity given the sensitive nature of abortion provision, and the use of snowball sampling. Future studies including nurses, trainees, and clinicians with greater racial and ethnic diversity are necessary to illuminate differences across these roles and identities and would improve the generalizability of findings. We chose to include only clinicians whose professional standards are guided by a formal code of medical ethics; future research should include administrative staff and medical assistants.

The MDT may not capture the nuances and changing nature of moral distress. While the MDT can indicate relative moral distress, no specific number has been validated as a predictor of burnout or attrition. Our modified MDT queried moral distress “as a result of the *Dobbs* decision,” which may have overly primed responses. Also, the Guttmacher Institute and WeCount categories of state-level restrictiveness do not reflect institutional restrictions; individuals experiencing moral distress based on institutional restrictions could have been misclassified. Moral distress among clinicians providing abortion should also be studied using qualitative methods.

## Conclusions

In this purposive, national survey study of clinicians providing abortion, moral distress was elevated among all clinicians and was more than twice as high among clinicians practicing in states that restrict abortion compared with those in states that protect abortion. These findings suggest that structural change that addresses bans on necessary health care is needed at institutional, state, and federal policy levels, including minimizing institutional barriers, bolstering state protections through abortion shield laws, and codifying federal protections for abortion.

## Notes

Supplement 1. **eAppendix.** Moral Distress Survey

Supplement 2.
Data Sharing Statement
