# Supplementary material for: State Abortion Policy and Moral Distress Among Clinicians Providing Abortion After the Dobbs Decision
Source: JAMA Netw Open. 2024 Aug 1;7(8):e2426248. doi: 10.1001/jamanetworkopen.2024.26248 (PMC11294965; doi:10.1001/jamanetworkopen.2024.26248)
Supplement: Supplement 1. — eAppendix. Moral Distress Survey [file jamanetwopen-e2426248-s001.pdf]

Supplemental Online Content

Rivlin K, Bornstein M, Wascher J, Turner AN, Norris AH, Howard D. State abortion policy and moral distress among clinicians providing abortion after the *Dobbs* decision. *JAMA Netw Open.* 2024;7(8):e2426248. doi:10.1001/jamanetworkopen.2024.26248

eAppendix. Moral Distress Survey

This supplemental material has been provided by the authors to give readers additional information about their work.

© 2024 Rivlin K et al. *JAMA Network Open.*

Moral Distress Survey

Please complete the survey below.

Thank you!

How old are you?

__________________________ (years old)

Which of the following best describes your race? (Choose ALL that apply)

- ☐ Black or African American
- ☐ White
- ☐ Asian or Asian American
- ☐ Native American, Alaska Native, or American Indian
- ☐ Native Hawaiian or Pacific Islander
- ☐ Other: ______
- ☐ Don't know
- ☐ Prefer not to answer

Are you Hispanic, Latino, or Latinx?

- ◯ Yes
- ◯ No
- ◯ Don't know
- ◯ Prefer not to answer

How would you describe your gender? (Choose ALL that apply)

- ☐ Woman
- ☐ Man
- ☐ Transgender Woman
- ☐ Transgender Man
- ☐ Non-binary
- ☐ Gender fluid
- ☐ Other: please specify below ______
- ☐ Don't know
- ☐ Prefer not to answer

How many years have you been providing abortion care?

__________________________ (years)

Are you currently in training (such as in residency, fellowship, or clinicals)?

- ◯ yes
- ◯ no
- ◯ prefer not to answer

Currently, what stage are you in your training?

- ◯ Residency
- ◯ Fellowship
- ◯ Clinicals
- ◯ Other: ______
- ◯ Don't know
- ◯ Prefer not to answer

What is your role in healthcare? (choose one)

- ◯ Physician
- ◯ Nurse
- ◯ Advanced Practice Provider
- ◯ Don't know
- ◯ Prefer not to answer

What kind of physician are you? (choose one)

- ◯ Ob/Gyn
- ◯ Family Medicine
- ◯ Other: ______
- ◯ Don't know
- ◯ Prefer not to answer

© 2024 Rivlin K et al. *JAMA Network Open.*

Have you completed a Complex Family Planning Fellowship? (Choose ALL that apply)	☐ Yes<br>☐ No<br>☐ I am currently completing a Complex Family Planning Fellowship<br>☐ I am planning on completing a Complex Family Planning Fellowship<br>☐ Don't know<br>☐ Prefer not to answer
Are you board certified in Complex Family Planning? (Choose all that apply)	☐ Yes<br>☐ No and I do not plan on taking Complex Family Planning boards<br>☐ No but I plan on taking Complex Family Planning boards<br>☐ I am not eligible to take the Complex Family Planning boards<br>☐ Don't know<br>☐ Prefer not to answer
What kind of Advanced Practice Provider are you? (Choose one)	◯ Nurse Practitioner<br>◯ Physician Assistant<br>◯ Certified Nurse Midwife<br>◯ Certified Registered Nurse Anesthetist<br>◯ Other: ______<br>◯ Don't know<br>◯ Prefer not to answer

© 2024 Rivlin K et al. *JAMA Network Open.*

Now we will ask you some questions about your abortion care practice. When we use the words "Dobbs decision" in this survey, we are referring to the Supreme Court of the United States decision in Dobbs v Jackson Women's Health Organization from June 24, 2022.

Are you currently engaged in abortion provision?

◯ yes
◯ no
◯ prefer not to answer

Currently, what percentage of your own clinical practice includes abortion care?

0% 50% 100%

(Place a mark on the scale above)

© 2024 Rivlin K et al. *JAMA Network Open.*

In what state or states do you currently provide abortion care? (Check ALL that apply)

☐ AL : Alabama
☐ AK : Alaska
☐ AZ : Arizona
☐ AR : Arkansas
☐ CA : California
☐ CO : Colorado
☐ CT : Connecticut
☐ DC : District of Columbia
☐ DE : Delaware
☐ FL : Florida
☐ GA : Georgia
☐ HI : Hawaii
☐ ID : Idaho
☐ IL : Illinois
☐ IN : Indiana
☐ IA : Iowa
☐ KS : Kansas
☐ KY : Kentucky
☐ LA : Louisiana
☐ ME : Maine
☐ MD : Maryland
☐ MA : Massachusetts
☐ MI : Michigan
☐ MN : Minnesota
☐ MS : Mississippi
☐ MO : Missouri
☐ MT : Montana
☐ NE : Nebraska
☐ NV : Nevada
☐ NH : New Hampshire
☐ NJ : New Jersey
☐ NM : New Mexico
☐ NY : New York
☐ NC : North Carolina
☐ ND : North Dakota
☐ OH : Ohio
☐ OK : Oklahoma
☐ OR : Oregon
☐ PA : Pennsylvania
☐ RI : Rhode Island
☐ SC : South Carolina
☐ SD : South Dakota
☐ TN : Tennessee
☐ TX : Texas
☐ UT : Utah
☐ VT : Vermont
☐ VA : Virginia
☐ WA : Washington
☐ WV : West Virginia
☐ WI : Wisconsin
☐ WY : Wyoming
☐ Outside of the United States
☐ Other
☐ Don't know
☐ Prefer not to answer

© 2024 Rivlin K et al. *JAMA Network Open.*

What state is the PRIMARY setting in which you currently provide abortion care? (Choose ONLY one)

◯ AL : Alabama
◯ AK : Alaska
◯ AZ : Arizona
◯ AR : Arkansas
◯ CA : California
◯ CO : Colorado
◯ CT : Connecticut
◯ DC : District of Columbia
◯ DE : Delaware
◯ FL : Florida
◯ GA : Georgia
◯ HI : Hawaii
◯ ID : Idaho
◯ IL : Illinois
◯ IN : Indiana
◯ IA : Iowa
◯ KS : Kansas
◯ KY : Kentucky
◯ LA : Louisiana
◯ ME : Maine
◯ MD : Maryland
◯ MA : Massachusetts
◯ MI : Michigan
◯ MN : Minnesota
◯ MS : Mississippi
◯ MO : Missouri
◯ MT : Montana
◯ NE : Nebraska
◯ NV : Nevada
◯ NH : New Hampshire
◯ NJ : New Jersey
◯ NM : New Mexico
◯ NY : New York
◯ NC : North Carolina
◯ ND : North Dakota
◯ OH : Ohio
◯ OK : Oklahoma
◯ OR : Oregon
◯ PA : Pennsylvania
◯ RI : Rhode Island
◯ SC : South Carolina
◯ SD : South Dakota
◯ TN : Tennessee
◯ TX : Texas
◯ UT : Utah
◯ VT : Vermont
◯ VA : Virginia
◯ WA : Washington
◯ WV : West Virginia
◯ WI : Wisconsin
◯ WY : Wyoming
◯ Outside of the United States
◯ Other
◯ Don't know
◯ Prefer not to answer

In what settings do you currently provide abortion care? (Choose ALL that apply)	☐ Community Hospital<br>☐ Academic Hospital<br>☐ State Funded Institution<br>☐ Planned Parenthood<br>☐ Independent free standing abortion clinic (excluding Planned Parenthood)<br>☐ Telehealth<br>☐ Other: ______<br>☐ Don't know<br>☐ Prefer not to answer
What is the PRIMARY setting in which you provide abortion care? (Choose ONLY one)	○ Community Hospital<br>○ Academic Hospital<br>○ State Funded Institution<br>○ Planned Parenthood<br>○ Independent free standing abortion clinic (excluding Planned Parenthood)<br>○ Telehealth<br>○ Other<br>○ Don't know<br>○ Prefer not to answer
Were you providing abortion care in the year leading up to the Dobbs decision (ie. anytime between May 2021 and May 2022)?	○ Yes ○ No ○ Prefer not to answer
In the year prior to the Dobbs decision, what percentage of your own clinical practice included abortion care?	0% 50% 100%<br>*(Place a mark on the scale above)*

© 2024 Rivlin K et al. *JAMA Network Open.*

In what state or states did you provide abortion care in the year prior to the Dobbs decision?

☐ AL : Alabama
☐ AK : Alaska
☐ AZ : Arizona
☐ AR : Arkansas
☐ CA : California
☐ CO : Colorado
☐ CT : Connecticut
☐ DC : District of Columbia
☐ DE : Delaware
☐ FL : Florida
☐ GA : Georgia
☐ HI : Hawaii
☐ ID : Idaho
☐ IL : Illinois
☐ IN : Indiana
☐ IA : Iowa
☐ KS : Kansas
☐ KY : Kentucky
☐ LA : Louisiana
☐ ME : Maine
☐ MD : Maryland
☐ MA : Massachusetts
☐ MI : Michigan
☐ MN : Minnesota
☐ MS : Mississippi
☐ MO : Missouri
☐ MT : Montana
☐ NE : Nebraska
☐ NV : Nevada
☐ NH : New Hampshire
☐ NJ : New Jersey
☐ NM : New Mexico
☐ NY : New York
☐ NC : North Carolina
☐ ND : North Dakota
☐ OH : Ohio
☐ OK : Oklahoma
☐ OR : Oregon
☐ PA : Pennsylvania
☐ RI : Rhode Island
☐ SC : South Carolina
☐ SD : South Dakota
☐ TN : Tennessee
☐ TX : Texas
☐ UT : Utah
☐ VT : Vermont
☐ VA : Virginia
☐ WA : Washington
☐ WV : West Virginia
☐ WI : Wisconsin
☐ WY : Wyoming
☐ Outside of the United States
☐ Other
☐ Don't know
☐ Prefer not to answer

© 2024 Rivlin K et al. *JAMA Network Open.*

What state was the PRIMARY state in which you provided abortion care in the year prior to the Dobbs decision? (Choose ONLY one)

◯ AL : Alabama
◯ AK : Alaska
◯ AZ : Arizona
◯ AR : Arkansas
◯ CA : California
◯ CO : Colorado
◯ CT : Connecticut
◯ DC : District of Columbia
◯ DE : Delaware
◯ FL : Florida
◯ GA : Georgia
◯ HI : Hawaii
◯ ID : Idaho
◯ IL : Illinois
◯ IN : Indiana
◯ IA : Iowa
◯ KS : Kansas
◯ KY : Kentucky
◯ LA : Louisiana
◯ ME : Maine
◯ MD : Maryland
◯ MA : Massachusetts
◯ MI : Michigan
◯ MN : Minnesota
◯ MS : Mississippi
◯ MO : Missouri
◯ MT : Montana
◯ NE : Nebraska
◯ NV : Nevada
◯ NH : New Hampshire
◯ NJ : New Jersey
◯ NM : New Mexico
◯ NY : New York
◯ NC : North Carolina
◯ ND : North Dakota
◯ OH : Ohio
◯ OK : Oklahoma
◯ OR : Oregon
◯ PA : Pennsylvania
◯ RI : Rhode Island
◯ SC : South Carolina
◯ SD : South Dakota
◯ TN : Tennessee
◯ TX : Texas
◯ UT : Utah
◯ VT : Vermont
◯ VA : Virginia
◯ WA : Washington
◯ WV : West Virginia
◯ WI : Wisconsin
◯ WY : Wyoming
◯ Outside of the United States
◯ Other
◯ Don't know
◯ Prefer not to answer

© 2024 Rivlin K et al. *JAMA Network Open.*

In what setting did you provide abortion care in the year prior to the Dobbs decision? (Choose ALL that apply)	☐ Community Hospital<br>☐ Academic Hospital<br>☐ State Funded Institution<br>☐ Planned Parenthood<br>☐ Independent free standing abortion clinic (excluding Planned Parenthood)<br>☐ Telemedicine<br>☐ Other<br>☐ Don't know<br>☐ Prefer not to answer
Please specify the setting in which you provided abortion care in the year prior to the Dobbs decision:	________________________________
What was the PRIMARY setting in which you provided abortion care in the year prior to the Dobbs decision? (Choose ONLY one)	◯ Community Hospital<br>◯ Academic Hospital<br>◯ State Funded Institution<br>◯ Planned Parenthood<br>◯ Independent free standing abortion clinic (excluding Planned Parenthood)<br>◯ Telemedicine<br>◯ Other<br>◯ Don't know<br>◯ Prefer not to answer

Now we are going to ask you some questions about your experience of Moral Distress in your clinical practice.

Moral distress occurs when you believe you know the ethically correct thing to do, but something or someone restricts your ability to pursue the right course of action.

Here is a moral distress thermometer:

© 2024 Rivlin K et al. *JAMA Network Open.*

Please select again from the following list ONE of the US states in which you practice and keep this clinical setting in mind when you answer the following questions.

For those who practice in more than one US state, you will have the option to answer these questions more than once (up to three times). For those who practice only in one US state, you will answer these questions once.

◯ AL : Alabama ◯ AK : Alaska
◯ AZ : Arizona ◯ AR : Arkansas
◯ CA : California ◯ CO : Colorado
◯ CT : Connecticut ◯ DC : District of Columbia ◯ DE : Delaware
◯ FL : Florida ◯ GA : Georgia
◯ HI : Hawaii ◯ ID : Idaho
◯ IL : Illinois ◯ IN : Indiana
◯ IA : Iowa ◯ KS : Kansas
◯ KY : Kentucky ◯ LA : Louisiana
◯ ME : Maine ◯ MD : Maryland
◯ MA : Massachusetts ◯ MI : Michigan
◯ MN : Minnesota ◯ MS : Mississippi
◯ MO : Missouri ◯ MT : Montana
◯ NE : Nebraska ◯ NV : Nevada
◯ NH : New Hampshire
◯ NJ : New Jersey ◯ NM : New Mexico
◯ NY : New York ◯ NC : North Carolina
◯ ND : North Dakota ◯ OH : Ohio
◯ OK : Oklahoma ◯ OR : Oregon
◯ PA : Pennsylvania ◯ RI : Rhode Island
◯ SC : South Carolina ◯ SD : South Dakota
◯ TN : Tennessee ◯ TX : Texas
◯ UT : Utah ◯ VT : Vermont
◯ VA : Virginia ◯ WA : Washington
◯ WV : West Virginia ◯ WI : Wisconsin
◯ WY : Wyoming ◯ Outside of the United States ◯ Other ◯ Don't know
◯ Prefer not to answer

Please select the NUMBER (0 to 10) on the Moral Distress Thermometer that best describes how much moral distress you are experiencing related to your abortion care practice (or lack of abortion care practice) in the US state you selected.

10 means that you are experiencing extreme distress regarding to your abortion care practice (or lack of abortion care practice) and 0 means that you are experiencing no distress regarding your abortion care practice.

0 5 10

(Place a mark on the scale above)

Which of the following describes your level of moral distress since the Dobbs decision in the US state you selected?

◯ I have been experiencing MORE moral distress since the Dobbs decision.
◯ I have been experiencing LESS moral distress since the Dobbs decision.
◯ My experience of moral distress has stayed THE SAME since the Dobbs decision.

Besides any changes to your abortion care practice that happened at the time of the Dobbs decision, is there another federal, state, local, or institutional policy that has most contributed to your experience of moral distress in your abortion care practice (or lack of abortion care practice) in the US state you selected?

◯ Yes ◯ No ◯ Don't know
◯ Prefer not to answer
◯ Not Applicable

© 2024 Rivlin K et al. *JAMA Network Open.*

Please briefly describe or name the federal, state, local, or institutional policy besides the Dobbs decision that has most contributed to your experience of moral distress and when this policy change occurred. (You can choose to leave this box blank)	__________________________________
Since the policy change that you descibe above, have you been experiencing more or less moral distress?	◯ I have been experiencing MORE moral distress.<br>◯ I have been experiencing LESS moral distress.<br>◯ My experience of moral distress has stayed THE SAME.

© 2024 Rivlin K et al. *JAMA Network Open.*

Now we are going to ask you questions about how your abortion care practice has changed since the Dobbs decision in the US state you selected.

Has abortion care become severely restricted or no longer legal in the US state you selected?	◯ Yes<br>◯ No<br>◯ Don't know<br>◯ Prefer not to answer<br>◯ Not applicable
Has abortion care become more accessible and less restricted in the US state you selected?	◯ Yes<br>◯ No<br>◯ Don't know<br>◯ Prefer not to answer<br>◯ Not Applicable
Are you seeing more patients in your practice seeking abortion care in the US state you selected?	◯ Yes<br>◯ No<br>◯ Don't know<br>◯ Prefer not to answer<br>◯ Not Applicable
Has a healthcare facility where you used to provide abortion care closed or stopped providing abortion care in the US state you selected?	◯ Yes<br>◯ No<br>◯ Don't know<br>◯ Prefer not to answer<br>◯ Not Applicable
Have you reduced your abortion care practice or stopped providing abortion care because of concerns that you will face legal or professional consequences for providing abortion care in the US state you selected?	◯ Yes<br>◯ No<br>◯ Don't know<br>◯ Prefer not to answer<br>◯ Not Applicable
Have you reduced your abortion care practice or stopped providing abortion care because of concerns related to your safety and/or the safety of family members/housemates in the US state you selected?	◯ Yes<br>◯ No<br>◯ Don't know<br>◯ Prefer not to answer<br>◯ Not Applicable
Have you retired from healthcare in the US state you selected?	◯ Yes<br>◯ No<br>◯ Don't know<br>◯ Prefer not to answer<br>◯ Not Applicable
Has your practice changed for reasons unrelated to abortion laws in the US state you selected?	◯ Yes<br>◯ No<br>◯ Don't know<br>◯ Prefer not to answer<br>◯ Not Applicable
Please specify the way your practice has changed:	________________________________

© 2024 Rivlin K et al. *JAMA Network Open.*

Has your practice changed for another reason not listed above in the US state you selected?	◯ Yes<br>◯ No<br>◯ Don't know<br>◯ Prefer not to answer<br>◯ Not Applicable
Please specify the unlisted reason that your practice has changed:	__________________________________
Would you like to answer the above questions related to moral distress and changes to your practice for another US state?	◯ Yes ◯ No

© 2024 Rivlin K et al. *JAMA Network Open.*

Ok, great! Please select again from the following list ONE of the US states in which you practice and keep this clinical setting in mind when you answer the following questions.

For those who practice in more than one US state, you will have the option to answer these questions up to three times. For those who practice only in one US state, you don't need to answer these questions again.

◯ AL : Alabama ◯ AK : Alaska
◯ AZ : Arizona ◯ AR : Arkansas
◯ CA : California ◯ CO : Colorado
◯ CT : Connecticut ◯ DC : District of Columbia ◯ DE : Delaware
◯ FL : Florida ◯ GA : Georgia
◯ HI : Hawaii ◯ ID : Idaho
◯ IL : Illinois ◯ IN : Indiana
◯ IA : Iowa ◯ KS : Kansas
◯ KY : Kentucky ◯ LA : Louisiana
◯ ME : Maine ◯ MD : Maryland
◯ MA : Massachusetts ◯ MI : Michigan
◯ MN : Minnesota ◯ MS : Mississippi
◯ MO : Missouri ◯ MT : Montana
◯ NE : Nebraska ◯ NV : Nevada
◯ NH : New Hampshire
◯ NJ : New Jersey ◯ NM : New Mexico
◯ NY : New York ◯ NC : North Carolina
◯ ND : North Dakota ◯ OH : Ohio
◯ OK : Oklahoma ◯ OR : Oregon
◯ PA : Pennsylvania ◯ RI : Rhode Island
◯ SC : South Carolina ◯ SD : South Dakota
◯ TN : Tennessee ◯ TX : Texas
◯ UT : Utah ◯ VT : Vermont
◯ VA : Virginia ◯ WA : Washington
◯ WV : West Virginia ◯ WI : Wisconsin
◯ WY : Wyoming ◯ Outside of the United States ◯ Other ◯ Don't know
◯ Prefer not to answer

Now we are going to ask you again some questions about your experience of Moral Distress in the US State you selected above.

Just as a reminder: Moral distress occurs when you believe you know the ethically correct thing to do, but something or someone restricts your ability to pursue the right course of action.

Here is a moral distress thermometer:

Please select the NUMBER (0 to 10) on the Moral Distress Thermometer that best describes how much moral distress you are experiencing related to your abortion care practice (or lack of abortion care practice) in the US state you selected.

10 means that you are experiencing extreme distress regarding to your abortion care practice (or lack of abortion care practice) and 0 means that you are experiencing no distress regarding your abortion care practice.

0 5 10

(Place a mark on the scale above)

Which of the following describes your level of moral distress since the Dobbs decision in the US state you selected?

◯ I have been experiencing MORE moral distress since the Dobbs decision.
◯ I have been experiencing LESS moral distress since the Dobbs decision.
◯ My experience of moral distress has stayed THE SAME since the Dobbs decision.

© 2024 Rivlin K et al. *JAMA Network Open.*

Besides any changes to your abortion care practice that happened at the time of the Dobbs decision, is there another federal, state, local, or institutional policy that has most contributed to your experience of moral distress in your abortion care practice (or lack of abortion care practice) in the US state you selected?	◯ Yes ◯ No ◯ Don't know<br>◯ Prefer not to answer<br>◯ Not Applicable
Please briefly describe or name the federal, state, local, or institutional policy besides the Dobbs decision that has most contributed to your experience of moral distress and when this policy change occurred. (You can choose to leave this box blank)	________________________________

© 2024 Rivlin K et al. *JAMA Network Open.*

**Now we are going to ask you questions about how your abortion care practice has changed since the Dobbs decision in the US state you selected.**	
Since the policy change that you descibe above, have you been experiencing more or less moral distress in the US state you selected?	◯ I have been experiencing MORE moral distress.<br>◯ I have been experiencing LESS moral distress.<br>◯ My experience of moral distress has stayed THE SAME.
Has abortion care become severely restricted or no longer legal in the US state you selected?	◯ Yes<br>◯ No<br>◯ Don't know<br>◯ Prefer not to answer<br>◯ Not applicable
Has abortion care become more accessible and less restricted in the US state you selected?	◯ Yes<br>◯ No<br>◯ Don't know<br>◯ Prefer not to answer<br>◯ Not Applicable
Are you seeing more patients in your practice in the US state you selected seeking abortion care?	◯ Yes<br>◯ No<br>◯ Don't know<br>◯ Prefer not to answer<br>◯ Not Applicable
Has a healthcare facility where you used to provide abortion care in the US state you selected closed or stopped providing abortion care?	◯ Yes<br>◯ No<br>◯ Don't know<br>◯ Prefer not to answer<br>◯ Not Applicable
Have you reduced your abortion care practice or stopped providing abortion care because of concerns that you will face legal or professional consequences for providing abortion care in the US state you selected?	◯ Yes<br>◯ No<br>◯ Don't know<br>◯ Prefer not to answer<br>◯ Not Applicable
Have you reduced your abortion care practice or stopped providing abortion care because of concerns related to your safety and/or the safety of family members/housemates in the US state you selected?	◯ Yes<br>◯ No<br>◯ Don't know<br>◯ Prefer not to answer<br>◯ Not Applicable
Have you retired from healthcare in the US state you selected?	◯ Yes<br>◯ No<br>◯ Don't know<br>◯ Prefer not to answer<br>◯ Not Applicable
Has your practice changed for reasons unrelated to abortion laws in the US state you selected?	◯ Yes<br>◯ No<br>◯ Don't know<br>◯ Prefer not to answer<br>◯ Not Applicable
Please specify the way your practice has changed:	__________________________________

Has your practice changed for another reason not listed above in the US state you selected?	◯ Yes<br>◯ No<br>◯ Don't know<br>◯ Prefer not to answer<br>◯ Not Applicable
Please specify the unlisted reason that your practice has changed:	__________________________________
Would you like to answer the above questions related to moral distress and changes to your practice for another US state?	◯ Yes ◯ No

© 2024 Rivlin K et al. *JAMA Network Open.*

Okay, great! Please select again from the following list ONE of the US states in which you practice and keep this clinical setting in mind when you answer the following questions.

For those who practice in more than two US states, this will be the third and final setting that we will be asking about. For those who practice in one or two US states, you don't need to answer these questions again.

◯ AL : Alabama ◯ AK : Alaska
◯ AZ : Arizona ◯ AR : Arkansas
◯ CA : California ◯ CO : Colorado
◯ CT : Connecticut ◯ DC : District of Columbia ◯ DE : Delaware
◯ FL : Florida ◯ GA : Georgia
◯ HI : Hawaii ◯ ID : Idaho
◯ IL : Illinois ◯ IN : Indiana
◯ IA : Iowa ◯ KS : Kansas
◯ KY : Kentucky ◯ LA : Louisiana
◯ ME : Maine ◯ MD : Maryland
◯ MA : Massachusetts ◯ MI : Michigan
◯ MN : Minnesota ◯ MS : Mississippi
◯ MO : Missouri ◯ MT : Montana
◯ NE : Nebraska ◯ NV : Nevada
◯ NH : New Hampshire
◯ NJ : New Jersey ◯ NM : New Mexico
◯ NY : New York ◯ NC : North Carolina
◯ ND : North Dakota ◯ OH : Ohio
◯ OK : Oklahoma ◯ OR : Oregon
◯ PA : Pennsylvania ◯ RI : Rhode Island
◯ SC : South Carolina ◯ SD : South Dakota
◯ TN : Tennessee ◯ TX : Texas
◯ UT : Utah ◯ VT : Vermont
◯ VA : Virginia ◯ WA : Washington
◯ WV : West Virginia ◯ WI : Wisconsin
◯ WY : Wyoming ◯ Outside of the United States ◯ Other ◯ Don't know
◯ Prefer not to answer

Now we are going to ask you again some questions about your experience of Moral Distress in the US State you selected above.

Just as a reminder: Moral distress occurs when you believe you know the ethically correct thing to do, but something or someone restricts your ability to pursue the right course of action.

Here is a moral distress thermometer:

Please select the NUMBER (0 to 10) on the Moral Distress Thermometer that best describes how much moral distress you are experiencing related to your abortion care practice (or lack of abortion care practice) in the US state you have selected.

10 means that you are experiencing extreme distress regarding to your abortion care practice (or lack of abortion care practice) and 0 means that you are experiencing no distress regarding your abortion care practice.

0 5 10

(Place a mark on the scale above)

Which of the following describes your level of moral distress since the Dobbs decision in the US state you selected?

◯ I have been experiencing MORE moral distress since the Dobbs decision.
◯ I have been experiencing LESS moral distress since the Dobbs decision.
◯ My experience of moral distress has stayed THE SAME since the Dobbs decision.

© 2024 Rivlin K et al. *JAMA Network Open.*

Besides any changes to your abortion care practice that happened at the time of the Dobbs decision, is there another federal, state, local, or institutional policy that has most contributed to your experience of moral distress in your abortion care practice (or lack of abortion care practice) in the US state you selected?	◯ Yes ◯ No ◯ Don't know<br>◯ Prefer not to answer<br>◯ Not Applicable
Please briefly describe or name the federal, state, local, or institutional policy besides the Dobbs decision that has most contributed to your experience of moral distress and when this policy change occurred. (You can choose to leave this box blank)	__________________________________
Since the policy change that you descibe above, have you been experiencing more or less moral distress?	◯ I have been experiencing MORE moral distress.<br>◯ I have been experiencing LESS moral distress.<br>◯ My experience of moral distress has stayed THE SAME.

© 2024 Rivlin K et al. *JAMA Network Open.*

**Now we are going to ask you questions about how your abortion care practice has changed since the Dobbs decision in the US state you selected.**	
Has abortion care become severely restricted or no longer legal in the US state you selected?	◯ Yes<br>◯ No<br>◯ Don't know<br>◯ Prefer not to answer<br>◯ Not applicable
Are you seeing more patients seeking abortion care in your practice in the US state you selected?	◯ Yes<br>◯ No<br>◯ Don't know<br>◯ Prefer not to answer<br>◯ Not Applicable
Has a healthcare facility where you used to provide abortion care closed or stopped providing abortion care in the US state you selected?	◯ Yes<br>◯ No<br>◯ Don't know<br>◯ Prefer not to answer<br>◯ Not Applicable
Have you reduced your abortion care practice or stopped providing abortion care because of concerns that you will face legal or professional consequences for providing abortion care in the US state you selected?	◯ Yes<br>◯ No<br>◯ Don't know<br>◯ Prefer not to answer<br>◯ Not Applicable
Have you retired from healthcare in the US state you selected?	◯ Yes<br>◯ No<br>◯ Don't know<br>◯ Prefer not to answer<br>◯ Not Applicable
Has your practice changed for reasons unrelated to abortion laws in the US state you selected?	◯ Yes<br>◯ No<br>◯ Don't know<br>◯ Prefer not to answer<br>◯ Not Applicable
Please specify the way your practice has changed:	__________________________________
Has your practice changed for another reason not listed above in the US state you selected?	◯ Yes<br>◯ No<br>◯ Don't know<br>◯ Prefer not to answer<br>◯ Not Applicable
Please specify the unlisted reason that your practice has changed:	__________________________________
You have reached the end of the survey questions. We have a few optional open-ended questions below that you are welcome to but not expected to complete. Would you be interested in answering these questions?	◯ Yes<br>◯ No

© 2024 Rivlin K et al. *JAMA Network Open.*

Click here to be directed to a separate survey where you can share your email address for compensation purposes	◯ Take me to the compensation survey

© 2024 Rivlin K et al. *JAMA Network Open.*
