# Supplementary material for: State Abortion Policy and Moral Distress Among Clinicians Providing Abortion After the Dobbs Decision
Source: JAMA Netw Open. 2024 Aug 1;7(8):e2426248. doi: 10.1001/jamanetworkopen.2024.26248 (PMC11294965; doi:10.1001/jamanetworkopen.2024.26248)
Supplement: Supplement 2. — Data Sharing Statement [file jamanetwopen-e2426248-s002.pdf]

Data Sharing Statement

Rivlin. State Abortion Policy and Moral Distress Among Clinicians Providing Abortion After the Dobbs Decision. *JAMA Netw Open*. Published August 01, 2024.
doi:10.1001/jamanetworkopen.2024.26248

Data
Data available: No

Additional Information
Explanation for why data not available: Given the legal complexity of abortion care in the United States, provider data will not be shared
